# Outcome after hepatectomy-delirium as an independent predictor for mortality

**DOI:** 10.1186/1471-2253-13-4

**Published:** 2013-02-02

**Authors:** Dalila Veiga, Clara Luís, Daniela Parente, Fernando Abelha

**Affiliations:** 1 Department of Anesthesia, Hospital de São João, Porto, Portugal; 2 Anesthesiology and Perioperative Care Unit-Surgical Department of Faculty of Medicine, University of Porto, Alameda Professor Hernani Monteiro, 4200-319, Porto, Portugal

**Keywords:** Quality of life, Hepatectomy, Outcome, Mortality, Activities of daily living

## Abstract

**Background:**

Most studies that follow up hepatectomy cases are limited in scope to an investigation of mortality and morbidity rates or the costs and length of hospital stay. In this study the authors aimed to characterize the quality of life and to evaluate mortality and its determinants after hepatectomy.

**Methods:**

This prospective study was carried in a Post-Anaesthesia Care Unit (PACU) over 15 months, and 70 patients submitted to hepatectomy were enrolled. Demographic and peri-operative characteristics were evaluated for associations with mortality. At admission and 6 months after discharge, patients completed a Short Form-36 questionnaire (SF-36) and have their independence in Activities of Daily Living (ADL) was evaluated. Binary and multiple logistic regression analyses were used to evaluate of associations with mortality, and the Wilcoxon signed rank test was used to compare SF-36 scores before and after 6 months after hepatectomy.

**Results:**

The mortality rate was 19% at 6 months. Multivariate analysis identified postoperative delirium as an independent determinant for mortality. Six months after discharge, 46% patients stated that their health in general was better or much better than that 1 year previously. Six months after hepatectomy, patients had worse scores in the physical function domain of SF-36; however, scores for all the other domains did not differ. At this time point, patients were more dependent in instrumental ADL than before surgery (32% versus 7%, p = 0.027).

**Conclusion:**

This study identified postoperative delirium as an independent risk factor for mortality 6 months after hepatectomy. After 6 months, survivors were more dependent in instrumental ADL tasks and had worse scores in the physical function domain of SF-36.

## Background

Although some studies have documented the beneficial outcome after hepatectomy surgery, most have been limited in scope to mortality and morbidity rates, or cost and length of hospital stay (LOS) [1,2]. Few studies have examined the dependence of these patients, and how they perceive changes in their own health after this procedure. In addition, little is known about the extent and impact of these changes on patient outcome.

Health-related quality of life (HRQOL) is recognized as an important component in the outcome evaluation of survivors and is also an indicator of patients’ general health status [3]. It has even been suggested that studies on outcome after Intensive Care Unit (ICU) stay should include HRQOL measurements [4].

Several questionnaires have been validated for the study of HRQL [5-9], and most of the measures that have been used are multi-item scales. Some multiple-item scales provide a total score as well as generating subscales that provide information on particular aspects. The Short-Form General Health Survey (SF-36) was developed during the Medical Outcomes Study (MOS) to measure generic health concepts that are relevant across age, disease and treatment groups [10]. It is a valid, self-completed questionnaire covering all aspects of Health Related Quality of Life (HRQOL); the Sf-36 has been used for post discharge ICU patients and for studying groups with other diseases; in addiction, it shows excellent reliability and validity [10,11]. This questionnaire has also been culturally adapted to the Portuguese and validated in a study by Ferreira [12,13].

Low functional status is associated with higher surgical risk. Patients who have only minor or non-clinical predictors of surgical risk, but with poor functional capacity are recommended to undergo noninvasive testing prior to hepatectomy. The determination of functional outcome and the identification of predictors of survival and functional recovery after hepatectomy may be fundamental in terms of evaluating the risk for these patients and for promoting proper treatment. The ability to care for oneself and live independently is considered a measure of functional outcome after hospitalization [14]. Functional status refers to the level of involvement in activities and is often used as a synonym for performance in Activities of Daily Living (ADL) [15]. ADL appraisal scales consider functional and instrumental activities. Patients’ ability to handle such activities has been assessed by generic or disease-specific measures of physical functional status. Furthermore, Katz’s Activities of Daily Living Scale [15] and the Lawton Instrumental Activities of Daily Living [16] have been used to investigate critical care survivors.

The aim of the present study was to characterize the quality of life and to evaluate mortality and its determinants after hepatectomy.

## Methods

The Institutional Review Board of the Centro Hospitalar São João approved the study protocol, and written consent was obtained from all patients.

This prospective cohort study was carried out in the multidisciplinary Post-Anaesthesia Care Unit (PACU) at the 1124-bed Centro Hospitalar São João, a community teaching hospital in Porto, Portugal, over a period of 15 months, beginning in March 2009. The PACU has a 5-bed Surgical ICU.

All consecutive postoperative patients submitted to elective hepatectomy and who were admitted to the surgical ICU area of the PACU were enrolled in the study. Patients readmitted during the study period were enrolled in relation to the time of their first admission.

The following clinical variables were recorded on admission to the ICU: age, sex, body weight, height and the American Society of Anesthesiologists physical status (ASA-PS). At admission, the core temperature was also registered. The ICU and in-hospital LOS and mortality were also recorded for all patients, and the Score of Simplified Acute Physiology Score (SAPS) II [17] and Acute Physiology & Chronic Health Evaluation (APACHE) II [18] were calculated using standard methods.

Specifically, pre admission comorbidities and any history of ischaemic heart disease, congestive heart disease, cerebrovascular disease, hypertension, renal insufficiency, diabetes, or hyperlipidaemia were recorded. The presence of coexisting conditions was assessed using the secondary diagnoses of the International Classification of Diseases, Ninth Revision, Clinical Modification (ICD-9-CM). Adapting a classification scheme developed by Lee *et al*. [19], we calculated a Revised Cardiac Risk Index (RCRI) score for each patient, assigning 1 point for each risk factor.

Each patient admitted was evaluated prospectively for the diagnosis of delirium, using the Intensive Care Screening Checklist (ICDSC) [20]; this evaluation was conducted by research staff physicians and a bedside nurse. The scores were assigned to each ICU patient by a nurse during every shift, and patients were either categorized as not having delirium” (scores of 0–3) or having delirium (scores of 4 or higher). ICDSC assessments were carried out at least once every 8 h for the entire duration of patients’ stay at the PACU. All the patients yielding an ICDSC score of 4 or higher at least once were considered to have postoperative delirium.

### Medical outcome survey Short-Form 36 (SF-36)

The HRQOL was assessed by the Medical Outcomes Study Short-form Health Survey (SF-36) [11]. The SF-36 evaluates 8 health domains considered to be important for patient well-being and health status. These domains reflect physical health and mental health, and the effects of health on daily functioning. There is 1 unscaled item that addresses self-reported changes in the respondent’s health status during the past year.

The answers to the question in SF-36 about self-reported changes in health status (“compared to one year ago, how you would rate your health in general now?”) were classified in three categories, as better/much better, remained the same and worse/much worse than 1 year ago.

To minimize distress for the next of kin, each patient’s records were checked on the hospital information system after 6 months to ascertain whether they were still alive. A copy of a formal letter was sent to all known survivors accompanied by a return envelope and a validated Portuguese SF-36 self-report form [12,13]. This version of the SF-36 has been validated for the study population in the Porto area from where the subjects of this study were drawn [21].

At admission and 6 months after PACU discharge, patients were asked to answer the SF-36 questionnaire for evaluating their quality of life.

### Activities of Daily Living (ADL)

Functional capacity was evaluated before surgery and 6 months after PACU discharge. We used a questionnaire to evaluate functional capacities of patients according to their ability to undertake personal ADL (P-ADL) and instrumental ADL (I-ADL). This questionnaire was based on Katz’ Index of Independence in ADL [22] and Lawton IADL [16] scales. Answers were also categorized into 2 groups, i.e. able or unable to perform each activity or group of activities. Patients were classified by their ability to perform physical and psychosocial ADL, and the following 4 categories were possible: (a) I-ADL and P-ADL independent, (b) I-ADL dependent but P-ADL independent, (c) P-ADL dependent but I-ADL independent and (d) both P-ADL and I-ADL dependent.

ADL was evaluated by the same investigator who questioned patients in person before and 6 months after surgery. When it was not possible to question patients directly, patients were questioned over the telephone.

### Outcome

The outcome endpoints considered were: (1) functional capacity and ADL, i.e. patients were considered dependent if they were dependent in at least 1 I-ADL or P-ADL activity; (2) quality of life, i.e. quality of life was evaluated at admission and 6 months after PACU discharge; and (3) mortality, i.e. patients were considered survivors if they were alive 6 months after PACU discharge.

### Statistical methods

Descriptive analyses of variables were used to summarize data and the Mann-Whitney U test was used to compare continuous variables between 2 groups of subjects. The chi-square test or Fisher’s exact test were used to compare proportions between 2 groups of subjects.

To identify independent predictors of mortality we performed univariate analyses using simple binary logistic regression with an odds ratio (OR) and 95% confidence interval (CI) with the following independent variables: type of surgery, age, sex, Body Mass Index (BMI), ASA-PS, temperature at admission to the PACU, comorbidities, RCRI score, duration of anaesthesia, and severity of disease scores (APACHE II and SAPS II). All variables were deemed to be significant if *P* < 0.05.

Multiple regression binary logistic was used forcing all variables in the model in order to identify independent predictors of mortality at 6 months follow-up. In this model, all covariates with p < 0.05 in the univariate analyses were entered; because of high correlation and colinearity between APACHE II and SAPSII only one of the severity of disease scores (SAPS II) was entered in this analysis. Age and length of ICU stay were also entered in this analysis to adjust for potential confounders.

The related samples Wilcoxon signed rank test was used to compare SF-36 scores before surgery and 6 months after surgery.

SPSS for Windows version 16.0 (SPSS, Chicago, IL, USA) was used to analyze the data.

## Results

During the study period, 70 patients met the inclusion criteria. The characteristics of all patients enrolled in the study are given in Table 1.

**Table 1:** Patient characteristics and outcome

**Variable**	**All**	**Malignant**	**Benign**	**P**
	**(n =70)**	**(n = 58)**	**(n = 12)**	
**Age in years,median(IQR)**	**59(48–67)**	**61(52–69)**	**43 (35–52)**	**<0.001**^**c**^
Age group, n (%)				0.002^b^
<65 years	42 (60)	30 (52)	12 (100)	
65 years	28 (40)	28 (48)	0 (0)	
**Sex,n(%)**				**0.011**^**a**^
Male	35 (50)	33 (57)	2 (17)	
Female	35 (50)	25 (43)	10 (83)	
**ASA physical status, n (%)**				**<0.001**^**a**^
I/II	23 (33)	11 (19)	12 (100)	
III/IV	47 (67)	47 (81)	0 (0)	
Body Mass Index in Kg/m^2^, median (IQR)	25 (22–30)	26 (23–30)	22 (21–30)	0.163^b^
Type of anesthesia, n (%)				0.440^a^
General	59 (84)	48 (83)	11 (92)	
Combined general locorregional	11 (16)	10 (17)	1(8)	
Duration of anesthesia (minutes), median (IQR)	300 (268–360)	300 (270–360)	285 (245–315)	0.298^b^
Intraoperative fluid volume				
Crystalloids (L.), median (P25–P75)	4.0 (3.2 – 4.9)	4.0 (3.3 – 4.9)	3.0 (3.0 – 5.2)	0.174^b^
Colloids (L.), median (P25–P75)	0.5 (0 – 0.5)	0.5 (0 – 0.5)	0 (0 – 0.4)	0.083^b^
Erythrocytes (Unit), median (P25–P75)	0 (0 – 1.0)	0 (0 – 1.0)	0 (0 – 0.0)	0.163^b^
Fresh Frozen plasma (Unit), median (P25–P75)	0 (0–0)	0 (0–0)	0 (0–0)	0.295^b^
Platelets (Unit), median (P25–P75)	0 (0–0)	0 (0–0)	0 (0–0)	0.517^b^
Temperature at PACU admission (°C), median (IQR)	34.7 (33.9–35.5)	34.8 (33.9–35.6)	34.7 (33.5–35.2)	0.623^b^
**Hypertension,n (%)**	**37(53)**	**35(60)**	**2(17)**	**0.006**^**a**^
**Hyperlipidemia,n (%)**	**25(36)**	**24(41)**	**1(8)**	**0.030**^**a**^
Pulmonary obstructive disease, n (%)	8 (11)	8 (14)	0 (0)	0.172^a^
Ischemic heart disease, n (%)	6 (9)	6 (10)	0 (0)	0.244^a^
Congestive heart disease, n (%)	10 (14)	10 (17)	0 (0)	0.132^a^
Cerebrovascular disease, n (%)	3 (4)	3 (5)	0 (0)	0.778^a^
Renal insufficiency, n (%)	4 (6)	3(5)	1 (8)	0.668^a^
Insulin therapy for diabetes, n (%)	3 (4)	3(5)	0 (0)	0.421^a^
Total RCRI, n (%)				0.349^a^
≤2	66 (94)	54 (93)	12 (100)	
>2	4 (6)	4 (7)	0 (0)	
Katz scale, median (IQR)	0 (0–0)	0 (0–0)	0 (0–0)	0.424^b^
Dependency in I-ADL, n (%)	3 (4)	3 (5)	0 (0)	0.421^a^
Lawton I-ADL, median (IQR)	7 (7–7)	7 (7–7)	7 (7–7)	0.176^b^
Dependency in P-ADL, n (%)	7 (10)	7 (12)	0 (0)	0.205^a^
**SAPS II,median (P25–75)**	**24(15–37)**	**29(21–39)**	**7(0–13)**	**<0.001**^**b**^
**APACHE II, median (IQR)**	**7(5–11)**	**8(6–11)**	**4(3–6)**	**<0.001**^**b**^
AKI	7 (10)	6 (10)	1(8)	0.833^a^
**Delirium**	**17(24)**	**17(29)**	**0(0)**	**0.025**^**a**^
PACU length of stay (minutes), median (IQR)	21 (19–57)	21 (19–57)	31 (20–60)	0.501^a^
**Hospital length of stay (days), median(IQR)**	**10(7–15)**	**11(7–16)**	**8(7–11)**	**0.034**^**b**^
Mortality in PACU, n (%)	3(4)	3 (5)	0 (0)	0.421^a^
Mortality in hospital, n (%)	5 (7)	5 (9)	0 (0)	0.291^a^
**Mortality at 6 months follow-up**	**13(19)**	**13(22)**	**0(0)**	**0.011**^**a**^

^a^ Fisher’s exact test, ^b^ Mann-Whitney U test, IQR, interquartile range.

RCRI, Revised Cardiac Risk Index I-ADL, Instrumental Activities of Daily Living; P-ADL, Personal Activities of Daily Living; SAPS II, Simplified Acute Physiology Score, APACHE II Acute Physiology & Chronic Health Evaluation PACU, Post Anaesthesia Care Unit; IQR, interquartile range.

Fifty per cent were male. The median age was 59 years, the median SAPS II was 24, median APACHE II was 7 and the median LOS in the PACU was 21 hours. Five patients (7%) died during their hospital stay (Table 1).

Fifty-eight patients (83%) had surgery because of malignant disease: this comprised primary tumors in 19 patients (27%) and secondary tumors in 39 patients (56%). Twelve patients (17%) had surgery because of benign disease.

Patients who submitted to hepatectomy because of malignancy were older, were more likely to be male, had higher ASA physical status, received higher amounts of erythrocytes during surgery, had a higher incidence of hypertension and hyperlipidaemia, were more severely ill, had more frequently postoperative delirium, had longer LOS and had higher mortality at 6 months follow-up (Table 1).

Sixty-five patients were discharged from the hospital; 13 died before the 6 month evaluation (19% global mortality at the time of evaluation). Of the remaining 57 patients, 23% did not answer the questionnaires at the 6 months follow-up but were known to be alive.

The characteristics of survivors and non-survivors at 6 months of follow-up are presented in Table 2. All patients submitted to surgery because of benign disease have survived and the mortality rate for patients with a malignant disease was 22% (Fisher’s exact test, P = 0.066). Of the 57 survivors, 49% were men, and the median age was 57 years. The median SAPS II score for this group was 24, the median APACHE II was 7, and the median Hospital LOS was 10 days.

**Table 2:** Univariate analysis for determinants of survival after hepatectomy

**Variable**	**Nonsurvivors/survivors**	**OR(95%CI)**	**P(a)**
**n (%)or median(IQR)**	**n =13 n =57**		
Type of hepatectomy surgery			0.105 (b)
for malignant disease	13 (100) / 45 (78)	–	
for benign disease	0 (0) /12 (21)	1	
Age	65 (54 – 71) / 57 (44 – 66)	1.04 (0.99 – 1.10)	0.109
Gender			0.759
Female	6 (46) / 29 (51)	1	
Male	7 (54) / 28 (49)	0.83 (0.25 – 2.77)	
BMI, median	25 (22 – 31) /25 (22 – 29)	0.99 (0.88 – 1.13)	0.981
Type of anesthesia			0.424
General	10 (77) /49 (86)	1	
Combined general loco regional	3 (23) / 8 (14)	1.84 (0.41 – 8.16)	
Duration of anesthesia (minutes)	300 (255 – 395) / 300 (265–330)	1.01 (0.99 – 1.01)	0.161
ASA Physical status			0.443 (b)
I/II	0 (0) /23 (40)	1	
III/IV	13 (100) / 34 (60)	–	
Intraoperative fluid volume, median			
Crystalloids (L.)	4.2 (4.0– 5.0) / 4.0 (3.1 – 4.9)	1.26 (0.86 – 1.87)	0.239
Colloids (L.)	0.5 (0–1.0) / 0.5 (0 – 0.5)	1.39 (0.34 – 5.79)	0.649
** Erythrocytes(Unit)**	**1(0 – 3.0) /0(0–1)**	**2.92(1.25–6.81)**	**0.013**
Fresh Frozen plasma (Unit)	0 (0 – 0) / 0 (0 – 0)	1.44 (0.80 – 2.58)	0.221
Platelets (Unit)	0 (0 – 0) / 0 (0 – 0)	–	1
Temperature at admission	34.2 (33.3 – 35.1) / 35.0 (33.9 – 35.6)	0.72 (0.44 – 1.17)	0.184
Hypertension	8 (62) / 29 (51)	1.55 (0.45 – 5.30)	0.489
Hyperlipidaemia	7 (54) / 18 (32)	2.53 (0.74 – 8.61)	0.138
Pulmonary obstructive disease	2 (15) / 6 (11)	1.55 (0.28 – 8.70)	0.621
Ischemic heart disease	3 (23) / 3 (5)	5.40 (0.95 – 30.7)	0.057
Congestive heart disease	3 (23) / 7 (12)	2.14 (0.47 – 9.73)	0.324
Cerebrovascular disease	2 (15) /1 (2)	10.18 (0.85 – 122)	0.067
Insulin therapy for diabetes	2 (15) / 1 (2)	10.18 (0.85 – 122)	0.067
Renal insufficiency	1 (8) / 3 (5)	1.50 (0.14 – 15.7)	0.735
**RCRI**			**0**.**019**
**≤2**	**10 (77)/56(98)**	**1**	
**>2**	**3(23) / 1(2)**	**16.8(1.59–178)**	
**Delirium**	**9** (**69) /8(14)**	**13.78(3.42–55.60)**	**<0.001**
Previous score in Katz scale	0 (0 – 0) / 0 (0 – 0)	–	1
Previous score in Lawton scale	7 (7 – 7) / 7 (7 – 7)	–	1
**SAPS II,median**	**32.0(23.5–48.0) / 24.0(13.0–33.5)**	**1.07(1.02–1.12)**	**0.011**
**APACHE II**	**12.0(10.5–16.0) /7.0** (**4.0–9.0)**	**1.55(1.21–1.98)**	**<0.001**
Length of PACU stay (hours)	20 (18 – 80) / 21 (19 – 50)	1.01 (0.99 – 1.03)	0.276
Length of Hospital stay (days)	14 (7 – 17) / 10 (7 – 13)	1.00 (0.95 – 1.06)	0.906

IQR, Interquartil range; OR, Odds Ratio; CI, Confidence Interval.

a) P value (binary logistic regression); b) Fisher’s exact test.

BMI, Body mass index; ASA, American Society of anesthesiologists; RCRI, Revised cardiac risk index; PACU, Post Anesthesia Care Unit; SAPS, Simplified Acute Physiology Score; APACHE II Acute Physiology & Chronic Health Evaluation.

### Mortality

Univariate analysis identified the amount of intra-operative erythrocytes administered, RCRI, postoperative delirium, SAPS II and APACHE II as predictors for mortality at the 6 months follow-up (Table 2).

In the multivariate analyses (Table 3) after adjustment for univariate predictors (intra-operative erythrocytes administered, RCRI and SAPS II) and for age and LOS in the ICU, postoperative delirium was identified as an independent predictor of mortality at the 6 months follow-up.

**Table 3:** Multivariate regression analysis for predictors of mortality

***Variable***	***Simple OR(95% CI)***	***p***	Adjusted*OR(95%CI)	***P***^***a***^
Age	1.04 (0.99 – 1.10)	0.109	0.97 (0.89 – 1.05)	0.438
Length of PACU stay (minutes)	1.01 (0.99 – 1.03)	0.276	0.98 (0.95 – 1.01)	0.263
Erythrocytes (Unit)	2.92 (1.25 – 6.81)	0.013	3.06 (0.95 – 9.84)	0.061
RCRI		0.019		0.152
≤2	1		1	
>2	16.8 (1.59 – 178)		6.52 (0.50 – 84.45)	
**Delirium**	**13.78(3.42 – 55.60)**	**<0.001**	**9.33(1.35 **– **64.61)**	**0.024**
SAPS II	1.07 (1.02 – 1.12)	0.011	1.06 (0.98 – 1.15)	0.169

a) Logistic regression analysis with enter method was used.

SAPS, Simplified Acute Physiology Score; APACHE II, Acute Physiology and Chronic Health Evaluation; RCRI, Revised Cardiac Risk Index; OR; Odds Ratio; CI, Confidence Interval.

*Adjusted for age, length of PACU stay, erythrocytes, RCRI and SAPS II. All variables were forced into the model in logistic regression analysis.

### Functional capacity and ADL

Six months after discharge from the PACU, patients were more dependent than before surgery in at least 1 I-ADL according to the Lawton Score (32% *vs* 7%, p = 0.027) (Table 4).

**Table 4:** Dependency in activities of daily living

**Variable**	**Before surgery**	**6 months after surgery**	**P**
**Median(IQR)**		**Median(IQR)**	
	**(n = 44)**	**(n = 44)**		
Personal activities of daily living			
Katz scale	0 (0–0)	0 (0–0)	0.133
Dependency in P-ADL, n (%)	2 (5)	5 (11)	0.323
Instrumental activities of daily living			
Lawton scale	7.0 (7.0–7.0)	7.0 (5.0–7.0)	0.058
**Dependency in I**-**ADL,n (%)**	**3(7)**	**14(32)**	**0.027**

IQR, Interquartil range.

I-ADL, Instrumental Activities of Daily Living; P-ADL, Personal Activities of Daily Living.

We found no differences in the dependency in P-ADL and Lawton scores when comparing these variables before surgery and 6 months after discharge from the PACU.

#### Quality of life measures

For the quality of life assessment the response rate at the 6 months follow-up was 77% (13 patients were lost to follow-up and did not answer the questionnaire).

Six months after hepatectomy, 45.5% of patients stated that their health in general was better/much better on the day of testing than 1 year earlier. However, 15.9% considered it to be worse/much worse than 1 year previously. A total of 38.6% considered that it had remained the same.

At the 6 months follow-up, scores for all SF-36 domains, except for the physical function domain were similar to the scores obtained before surgery. For this domain, the scores observed 6 months after surgery were worse than those recorded before surgery (Table 5).

**Table 5:** **SF**-**36 means before and after hepatectomy**

**Variable**	**Before hepatectomy**	**After hepatectomy**	**P(a)**
	**(n = 44)**	**(n = 44)**	
**SF-36 domains**	**mean ± sd**	**mean ± sd**	
**Physical function**	**85.0 ± 20.1**	**71.4 ± 30.2**	**0.025**
Role physical	58.2 ± 35.6	63.4 ± 31.9	0.406
Bodily pain	67.5 ± 28.1	73.8 ± 30.6	0.326
General health perception	60.3 ± 21.1	60.5 ± 22.6	0.979
Vitality	43.4 ± 14.9	45.6 ± 14.7	0.472
Social functioning	72.4 ± 24.5	65.9 ± 28.1	0.286
Role emotional	68.6 ± 25.6	64.6 ± 30.7	0.463
Mental health	52.8 ± 19.4	59.1 ± 20.5	0.182

Wilcoxon signed rank test; SF-36, Short-form 36.

## Discussion

The principal findings of our study are as follows: (a) after hepatectomy, the development of postoperative delirium was considered an independent risk factor for mortality at 6 months after discharge from the PACU; (b) 6 months after surgery, patients were more dependent with regards to I-ADL; and (c) 6 months after hepatectomy the patients enrolled in this study had lower scores in the physical function domain of SF-36 indicating worse quality of life in this domain.

This study identified the following risk factors for mortality 6 months after hepatectomy: the amount of erythrocytes administered intraoperatively, RCRI, postoperative delirium and severity of disease scores.

A few studies have evaluated the effects of perioperative transfusions in morbidity and mortality after hepatectomy surgery. The amount of erythrocytes administered is associated with higher blood losses, larger resections, and increased technical difficulty [23]. In addiction, blood transfusions have been associated with higher tumor recurrence rates due to the immunosuppressive effects of these transfusions [23]. In fact, hepatectomy is associated with major perioperative blood loss. Several techniques have been advocated in order to reduce the amount of transfusions [24]. During inflow occlusion at the time of parenchyma resection, the main source of bleeding is backflow from the valveless hepatic veins and control of central and hepatic venous pressure has been documented as crucial to reduce the blood loss [24,25].

However, Kuroda *et al*. [26] recently published a retrospective analysis, which found that perioperative blood transfusion did not have any influence on survival and carcinoma recurrence after hepatectomy surgery. Conversely, other studies have shown that patients who received blood transfusions had higher morbidity rates [24,27].

In this study RCRI appears as a predictor of mortality at the 6 months follow-up. This RCRI, developed by Lee *et al*. [28], has been proposed as a cardiac risk prediction index based on clinical characteristics. However, although this score is validated and widely used for estimating the risk of a perioperative major adverse myocardial event other studies have linked higher RCRI with higher mortality rates [29][30].

APACHE II and SAPS II are two of the most commonly used scores for prediction of outcome of patients admitted to ICU: therefore, it is expected that patients with higher scores have higher mortality rates and worse associated outcomes; this in accordance with our results [31,32].

Delirium, or an “acute confusional state,” is a transient global disorder of cognition. The condition is associated with increased morbidity and mortality rates as well as longer LOS in the hospital, ICU and post anesthesia care units [33-41].

It is well known that mortality is associated with postoperative delirium development in a surgical ICU. This was documented by Leslie *et al*. [42] in a study that focused on delirium and outcome. Furthermore McCusker *et al*. calculated hazard ratios of 2.11 for mortality in delirious patients after 12 months[43]. Ely *et al*. [33] and Lin *et al*. [44] have also reported that delirium is an independent predictor of mortality in critical care patients. Indeed, according to their results, every day of delirium was associated with a 10% higher risk of death and worse long-term cognitive function [33]. However, to the best of our knowledge, our study is the first to indicate that delirium development is also an independent risk factor for mortality after hepatectomy.

In our study, postoperative delirium was considered an independent determinant for mortality at the 6 months follow-up. This was the case not only after adjustment for variables from the univariate analyses but also after adjustment for potential confounders such as age and LOS at the PACU. We have forced these variables in the multivariate analysis because the probability of postoperative delirium may be increased in elderly patients and in patients who stay longer in the PACU. In fact, age is the most consensual predictor for delirium and this has been widely reported [45]. Postoperative delirium has also been associated with longer LOS in the ICU and PACU [46].

The outcomes assessed in this study were mortality rate, quality of life and rate of dependency in P-ADL 6 months after hepatectomy. The time point for assessment of outcome (i.e. 6 months after discharge from the PACU) was chosen to minimize dropouts and to ensure that the recovery time was sufficient for a surgery such as hepatectomy. Moreover, we did not choose a period greater than 6 months in order to avoid the effects of chronic underlying conditions or the onset of new and unrelated health problems that could interfere with our outcomes [47].

In our study, at the 6 months evaluation, 46% of the patients stated that their general health was better/much better than 1 year earlier, and only 16% considered their health status it to be worse/much worse.

This shows a relatively high level of HR-QOL among an important percentage of those who survived 6 months after discharge from the PACU and these findings agree with those of other reports that used different tools [7,48]. This is important because according to SF-36 scores revealed that there were no differences in quality of life before and after surgery, except in the physical function domain.

After hepatectomy the patients enrolled in this study had worse results for the physical function domain, but the scores for all the other domains there were unchanged. This may be related to a difficulty in recovering in terms of physical activity after surgery. This drop in physical function scores might also be a reflection of the invasiveness of the procedure and may also explain the number of patients with disabilities encountered in ADL. It could also highlight the substantial strain of therapy imposed on patients who undergone hepatectomy.

This may also be indicative of an increased risk of developing limitations to quality of life of patients.

Our study has several limitations. Firstly, only a small number of patients were included in the analysis; thus, there were only a small number of participants at the follow-up and a limited number of mortality events. For this small patient population, we studied multiple variables and this may have increased statistical type II error. Even so, the sample may have been very small to detect other statistically significant factors. Secondly, the patients were only screened for delirium in the PACU. This may have increased the probability of developing delirium in patients with higher severity of disease and more comorbidities. Furthermore, patients may have developed delirium only after PACU discharge and development of postoperative delirium in the later stages may have been missed.

Thirdly, for the 6 month follow-up after PACU discharge, in those instances where face-to-face conversations where not possible, patients were asked over the telephone about their ADL performance. The possibility of cognitive impairment, which was not evaluated at that time, was not considered; this could also have had an impact on their answers to the SF-36 at that time.

## Conclusion

In conclusion, this study shows that the development of postoperative delirium is an independent risk factor for mortality at the 6 month follow-up. At 6 months after hepatectomy, survivors were more dependent in ADL tasks and had worse scores for the physical function domain of the SF-36 questionnaire.

## Competing interests

The authors did not use funds for the research and have no conflicts of interest.

## Authors’ contributions

All people listed as authors contributed to the preparation of the manuscript and no person or persons other than the authors listed have contributed significantly to its preparation. Each listed author participated in the work to the extent that they could all publicly defend its content. They all read the manuscript before its submission for publication and are prepared to sign a statement stating they had read the manuscript and agree to its publication. FA made all coordination of the study, performed statistical analyses and wrote the draft the manuscript. DV, CL, DP and FA participated in the design of the study, conceived of the study, and participated in its design. All authors read and approved the final manuscript.

## Name and address of the department where the work was performed

Post Anesthesia Care Unit at Centro Hospitalar São João

Department of Anesthesiology

Centro Hospitalar São João, Porto, Portugal

Alameda Professor Hernani Monteiro

4200-319 Porto, Portugal

## Pre-publication history

The pre-publication history for this paper can be accessed here:

http://www.biomedcentral.com/1471-2253/13/4/prepub
